# Treating C3 glomerulopathy with eculizumab

**DOI:** 10.1186/s12882-017-0802-4

**Published:** 2018-01-12

**Authors:** Thomas Welte, Frederic Arnold, Julia Kappes, Maximilian Seidl, Karsten Häffner, Carsten Bergmann, Gerd Walz, Elke Neumann-Haefelin

**Affiliations:** 1 Department of Nephrology, Medical Center–University of Freiburg, Germany, Hugstetter Strasse 55, 79106 Freiburg, Germany; 2 Department of Pneumology, Medical Center–University of Freiburg, Germany, Killianstrasse 4, 79106 Freiburg, Germany; 3 Department of Pathology, Medical Center–University of Freiburg, Germany, Breisacher Strasse 115A, 79106 Freiburg, Germany; 4 Department of Pediatrics and Adolescent Medicine, Medical Center–University of Freiburg, Germany, Heiliggeiststrasse 1, 79106 Freiburg, Germany; 5 Center for Human Genetics, Bioscientia, Ingelheim, Germany, Konrad-Adenauer-Strasse 17, 55218 Ingelheim, Germany

**Keywords:** C3 glomerulopathy, C3 glomerulonephritis, Dense deposit disease, Complement, Eculizumab

## Abstract

**Background:**

C3 glomerulopathy (C3G) is a rare, but severe glomerular disease with grim prognosis. The complex pathogenesis is just unfolding, and involves acquired as well as inherited dysregulation of the alternative pathway of the complement cascade. Currently, there is no established therapy. Treatment with the C5 complement inhibitor eculizumab may be a therapeutic option. However, due to rarity of the disease, parameters predicting treatment response remain largely unknown.

**Methods:**

Seven patients with C3G (five with C3 glomerulonephritis and two with dense deposit disease) were treated with eculizumab. Subjects underwent biopsy before enrollment. The histopathology, clinical data, and response to eculizumab treatment were analyzed. The key parameters to determine outcome were changes of serum creatinine and urinary protein over time.

**Results:**

After treatment with eculizumab, four subjects showed significantly improved or stable renal function and urinary protein. A positive response occurred between 2 weeks and 6 months after therapy initiation. One subject (with allograft recurrent C3 glomerulonephritis) initially showed a positive response, but relapsed when eculizumab was discontinued, and did not respond after re-initiation of treatment. Two subjects showed impaired renal function and increasing urinary protein despite therapy with eculizumab.

**Conclusions:**

Eculizumab may be a therapeutic option for a subset of C3G patients. The response to eculizumab is heterogeneous, and early as well as continuous treatment may be necessary to prevent disease progression. These findings emphasize the need for studies identifying genetic and functional complement abnormalities that may help to guide eculizumab treatment and predict response.

**Electronic supplementary material:**

The online version of this article (10.1186/s12882-017-0802-4) contains supplementary material, which is available to authorized users.

## Background

C3 glomerulopathy (C3G) is a recent description of a disease characterized by uncontrolled activation of the alternative complement pathway leading to predominantly glomerular deposition of complement C3 and C3 fragments [1–4] and characteristic histo-pathological features for membranoproliferative glomerulonephritis (MPGN). Based on electron microscopy analysis, C3G can be subclassified as dense deposit disease (DDD) and C3 glomerulonephritis (C3GN). A distinction is made to MPGN caused by immune complex mediated dysregulation of the classical complement cascade, although both disease spectra share common pathophysiological features [5]. Patients present with varying levels of urinary protein and/or hematuria. Concomitant loss of renal function leads to end-stage renal disease (ESRD) in many cases.

C3G is a rare disease with an estimated incidence of 1–2 cases per million [6]. Renal prognosis is poor with very few reports of spontaneous remission [7] and a vast majority of progression to ESRD with rates of up to 50% within a decade, and recurrence rates of 45–60% in allografts [8–12]. A cohort study with 80 patients identified age > 16 years, DDD subtype, and crescentic GN as predictors for ESRD [6].

Dysregulation of the alternative complement cascade plays a primary role in the pathogenesis of C3G and atypical hemolytic uremic syndrome (aHUS). Uncontrolled activation of the alternative complement pathway is driven by inherited mutations in complement proteins and cofactors (e.g. *C3, CFB, CFH, CFI, CFHR1–5*), or by acquired defects (e.g. CFH, CFB, C3 convertase autoantibodies) [13–16]. While aHUS results from dysregulation of complement factors on cell surfaces (the ‘solid phase’) [17] leading to global endothelial damage, C3G seems to occur after activation of the alternative complement pathway in the fluid phase [14, 15, 18].

In contrast to immune complex mediated MPGN, which is managed by treating underlying infections, autoimmune diseases, or cancer, treatment strategies in the setting of C3G are still evolving. So far, no generally effective treatment has been identified [19]. Treatment of C3G with immunosuppression (e.g. calcineurin inhibitors, cyclophosphamide, mycophenolate mofetil, or rituximab) [9, 20–23], plasma infusion [24], or plasmapheresis [25] has been described in small cohorts with inconclusive results. With the emerging role of complement in C3G, complement-modulating agents have added new therapeutic options. Eculizumab is a monoclonal humanized antibody that binds C5 and prevents assembly of the membrane attack complex (C5b-9) thereby blocking the final complement cascade. Several case reports and one open-label study showed a potential benefit of eculizumab for patients with C3G [22, 26–39].

In this study, we characterize clinical and pathological features of seven patients with C3G (five with C3GN, two with DDD), evaluate their treatment response to eculizumab, and discuss critical features of their individual therapeutic outcome.

## Methods

Data were collected from medical records. Inclusion criteria were age > 18 years, histopathological diagnosis of C3G, and treatment with eculizumab (Soliris; Alexion Pharmaceuticals, Cheshire, CT). Seven patients (five with C3GN, two with DDD) treated with eculizumab between 2013 and 2016 for C3G were enrolled in the study. Data were collected from the time of treatment initiation until last follow-up. Outcome measurements were change of kidney function, and urinary protein over time.

Positive treatment response was defined as >30% decrease of serum creatinine, urinary protein, or hematuria after 3 months of treatment. Stable disease was defined as serum creatinine, urinary protein, or hematuria between ±30% after 3 months of treatment. Negative treatment response was defined as increase >30% of serum creatinine, urinary protein, or hematuria after 3 months of treatment.

Treatment with Angiotensin converting enzyme inhibitors (ACEI), or angiotensin receptor blockers (ARB) was administered to all patients for blood pressure control and reduction of urinary protein. However, disease progression warranted for specific treatment in all patients.

All patients gave informed consent for eculizumab treatment. The study was approved by the Ethics Committee of the University Medical Center, Freiburg, Germany. Before eculizumab administration, all patients received meningococcal vaccinations. Eculizumab was given according to aHUS standard treatment protocol [40]. Briefly, eculizumab was administered weekly at a dose of 900 mg (week 1–4), followed by eculizumab every other week at a dose of 1200 mg (from week 5). No serious side effects were reported. Serum creatinine and urinary protein levels were monitored regularly. Reference ranges for serum creatinine levels were defined for men as 0.84–1.25 mg/dl, for women as 0.66–1.09 mg/dl. Reference ranges for urinary protein were defined as <0.15 g/g. Due to the small study population, no statistical analysis was performed. Data are presented for each individual case.

## Results

### Patient characteristics

The report includes five patients (all white Caucasians) with C3GN (two males and three females) and two patients with DDD (both female, Table 1). Three patients had recurring allograft C3G. One patient was diagnosed with de novo allograft C3GN after kidney transplantation with ESRD due to focal segmental glomerulosclerosis (FSGS). Four patients received immunosuppressive agents prior to eculizumab with varying success. The mean age at diagnosis was 29 years (range = 14–59 years). At diagnosis all subjects had either impaired renal function (mean serum creatinine 2 mg/dl; range = 0.8–3.3 mg/dl) and/or elevated levels of urinary protein (urinary protein to creatinine ratio [UPCR] 3.6 g/g; range = 0.3–5.4 g/g). All patients presented with varying levels of hematuria.

**Table 1:** Patient characteristics and treatment response

	*C3GN1*	*C3GN2*	*C3GN3*	*DDD1*	*C3GN4*	*DDD2*	*C3GN5*
native/graft^a^	graft	native	graft	native	graft	native	native
Baseline SCr (mg/dl)	1.9	2.3	2.1	1.5	3.3	2.1	0.8
Baseline UPCR (g/g)	5.4	0.3	0.3	5.0	3.4	5.4	5.6
Baseline hematuria^b^ (0–4)	4	4	3	4	4	4	2
Therapy	**pre Tx** none	none	**pre Tx** prednisone, MMF, azathioprine	none	**pre Tx** cyclo- sporine A, cyclophos-phamide	none	PEX, FFP, immune-globulins, prednisone, MMF [25]
	**post Tx** prednisone, tacrolimus, MMF, rituximab		**post Tx** prednisone, tacrolimus, MMF (graft)		**post Tx** prednisone, tacrolimus, MMF, cyclophos-phamide, PEX [41] (graft)		
Time to graft relaps (months)	48	–	3	–	de novo C3GN 135 months post Tx	–	–
Time diagnosis to eculizumab (months)	144 (native); 0 (graft)	7	88 (native); 2 (graft)	128	2 (graft)	7	11
Treatment duration (months)	27	26	9	24	3; 3	16	28
Response SCr	stable	yes	stable	stable	initially yes; recurrence no	initially stable; ultimately no	no
Response UPCR	yes	stable	stable	initially yes; ultimately stable	initially yes; recurrence no	initially stable; ultimately no	no
Response hematuria	stable	yes	stable	yes	stable	yes	yes

*Abbreviations*: *FFP* fresh frozen plasma, *MMF* mycophenolate mofetil, *PEX* plasmapheresis, *Tx* kidney transplantation

^a^native: applies to native kidney; graft: applies to kidney transplant

^b^hematuria measured by urine dipstic (scale from 0 to 4)

### Course of C3G disease

**Fig. 1:**
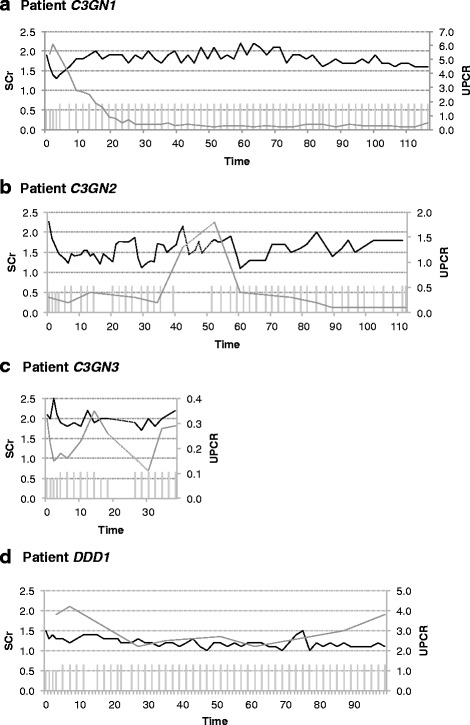
Positive response to eculizumab treatment in C3G patients. Graphs show treatment response to eculizumab. Column charts show applied eculizumab dose (low column: 900 mg; high column: 1200 mg). Line charts display serum creatinine levels (black; SCr; mg/dl), and urinary protein levels (grey; UPCR; g/g) over time (weeks). **a** Patient *C3GN1*. **b** Patient *C3GN2*. Dashed lines show the period of time (12 weeks) where eculizumab was paused. **c** Patient *C3GN3*. Dashed lines show the period of time (4 weeks) where eculizumab was paused. **d** Patient *DDD1*

*Patient C3GN1* was diagnosed with C3GN in his/her late thirties, 4 years after receiving an allogenic kidney transplant (baseline creatinine 1.6 mg/dl post transplantation; immunosuppression with tacrolimus, MMF [mycophenolate mofetil], and prednisone) due to ESRD of MPGN (initial diagnosis 11 years earlier with classification as MPGN type I). The patient presented with elevated levels of serum creatinine (1.9 mg/dl), urinary protein (5–6 g/g), and hematuria. The kidney biopsy revealed discrete glomerular fibrosis, mesangial hypercellularity, lymphocyte infiltration, and pronounced C3 staining in accordance with C3GN (Table 2). Complement assays showed complement activation with low C3 levels (Additional file 1: Table S2). Additionally, C3 convertase autoantibodies were detected (C3 nephritic factors, C3NeF; Table 2). Immunosuppression was maintained with prednisone, tacrolimus, and MMF. One-time administration of rituximab did neither affect creatinine levels nor urinary protein. Treatment with eculizumab over 27 months maintained stable kidney transplant function (serum creatinine levels of 1.6–2.2 mg/dl), greatly improved urinary protein (0.2–0.4 g/g), and led to stable hematuria (Fig. 1a). Complement testing at week 17 and 43 of treatment suggested effective complement blockade (CH50 < 10 IE/ml, ref. range 20–50 IE/ml; Additional file 1: Table S2).

**Table 2:** Histo-pathology, genetic and autoantibody testing

	*C3GN1*	*C3GN2*	*C3GN3*	*DDD1*	*C3GN4* 1^st^ biopsy	*C3GN4* 2^nd^ biopsy	*DDD2*	*C3GN5* 1^st^ biopsy	*C3GN5* 2^nd^ biopsy
Light microscopy									
Glomerula (*n*)	20	15	3	8	25	13	11	24	19
Global sclerosis (%)	5	7	0	0	56	38	9	4	16
Partial sclerosis (%)	0	0	0	0	0	15	91	13	0
IF/TA^a^ (%)	5	10	0	5	5	25	30	10	10
Crescents (%)	0	0	0	0	0	0	0	0	0
Mesangial proliferation	pos	pos	pos	pos	pos	pos	neg	pos	pos
Leukocyte infiltration^b^ (0–3)	2	2	1	0	1	2	0	1	0
Immunofluorescence^c^									
C3 (0–3)	3	2	3	2	2	3	2	3	3
C4d (0–3)	NT	NT	0	NT	0	0	NT	NT	NT
C5b-9 (0–3)	2	1	2	NT	1	1	1	NT	2
IgA (0–3)	1	1	0	1	0	0	1	0	0
IgG (0–3)	1	0	1	1	0	0	0	1	2, κ light chain, mouse pos
IgM (0–3)	1	2	1	1	1	1	2	1	1
Electron microscopy									
DDD deposits	neg	neg	neg	pos	neg	neg	pos	NT	neg
Genetic analysis									
*CFH*	NT	NT	neg	neg	neg		neg	neg	
*CFI*	NT	neg	neg	NT	HET c.1322A > G		neg	neg	
*CFB*	NT	NT	NT	NT	NT		neg	NT	
*CFHR1–5*	NT	NT	neg	NT	HET del *cfhr1, 3*		HET *CFHR5* c.832G > A	neg	
*MCP*	NT	neg	neg	NT	neg		neg	neg	
*C3*	NT	NT	HET c.2203C > T	neg	neg		neg	neg	
*COL4A3*	NT	NT	NT	NT	HET c.4484A > G		NT	NT	
Autoantibodies									
C3Nef	pos	pos	neg	neg	pos		neg	pos	
C3b	NT	pos	NT	NT	NT		NT	NT	
CFH	pos	pos	NT	NT	neg		neg	NT	
CFI	neg	NT	NT	NT	neg		NT	NT	

*Abbreviations*: *del* deletion, *HET* heterozygous, *NT* not tested

^a^interstitial fibrosis and tubular atrophy

^b^leukocyte infiltration was graded on a scale from 0 to 3

^c^antibody staining was graded on a scale from 0 to 3

*Patient C3GN2* with a history of HIV infection (HAART had been successfully applied to control HIV infection) presented with serum creatinine levels of 2.3 mg/dl, urinary protein levels of 0.3 g/g, and hematuria in his/her early forties. Kidney biopsy revealed global mesangial and endocapillary hypercellularity with discrete glomerular sclerosis. Immunofluorescence and electron microscopy revealed distinct C3 deposition with little to no immunoglobulin deposition (Table 2) leading to the diagnosis of C3GN. Complement analysis showed increased C3 turnover and sMAC formation (Additional file 1: Table S3). Antibody and genetic testing showed no pathologic mutations*,* but identified antibodies against C3NeF, C3b, and CFH (Table 2). Eculizumab treatment resulted in an improvement of renal function with slightly improved serum creatinine levels at 1.2–1.7 mg/dl and low range urinary protein (0.1–0.2 g/g; Fig. 1b). Notably, a short interruption of eculizumab treatment for 12 weeks led to a dramatic increase of urinary protein (maximum urinary protein levels 1.8 g/g), worsened renal function (maximum serum creatinine levels 2.2 mg/dl), and enhanced sMAC formation (maximum sMAC levels 914 ng/dl). Re-initiation of eculizumab promptly reduced urinary protein (0.1–0.3 g/g), restored kidney function (serum creatinine levels 1.4–1.8 mg/dl), and improved hematuria.

*Patient C3GN3* presented with relapsing C3GN in his/her mid-thirties, 3 months after receiving allogenic kidney transplantation (post transplantation creatinine 1.5 mg/dl; immunosuppression with tacrolimus, MMF, and prednisone) due to ESRD of C3GN (initial diagnosis 7 years earlier). Previous treatment approaches included prednisone, MMF, and azathioprine. The patient presented with elevated serum creatinine (2.1 mg/dl), discrete urinary protein (0.3 g/g), and hematuria (Additional file 1: Table S4). Transplant kidney biopsy revealed no signs of graft rejection, but showed mesangial proliferation, lymphocyte infiltration and predominant C3 staining without immunoglobulin deposition (Table 2). Electron microscopy confirmed relapsing C3GN. Antibody and genetic testing revealed neither autoantibodies nor pathophysiological relevant mutations in any of the known disease genes (Table 2). Notably a heterozygous variant change of questionable pathophysiological relevance in exon 17 of *C3* was detected (detailed discussion Additional file 1: Text S1). Treatment with eculizumab stabilized kidney function (serum creatinine 1.9–2.2 mg/dl), urinary protein (0.25–0.35 g/g), and hematuria (Fig. 1c). Immunosuppression was maintained with prednisone, tacrolimus, and MMF. Of note, eculizumab had to be paused for 4 weeks due to immunosuppression-induced agranulocytosis.

*Patient DDD1* presented in his/her early twenties with impaired renal function (serum creatinine 1.5 mg/dl), nephrotic range urinary protein (5.7 g/g), and hematuria. Complement analysis showed activation of the alternative pathway and high sMAC levels (Additional file 1: Table S5). A kidney biopsy at the department of pediatric nephrology 10 years before had shown mesangial proliferation, and prominent C3 staining with little immunoglobulin staining. Electron microscopy confirmed DDD (Table 2). Antibody analysis and genetic testing identified neither autoantibodies against C3NeF, nor pathophysiological relevant mutations in *CFH* (Table 2). Treatment with eculizumab stabilized kidney function (serum creatinine 1.1–1.3 mg/dl) and initially improved urinary protein (2.2–2.7 g/g). Hematuria levels significantly decreased. However, urinary protein deteriorated after 87 weeks of therapy (3.0–3.7 g/g) (Fig. 1d). Blood pressure was carefully controlled and within normal range.

*Patient C3GN4* presented with de novo C3GN at the in his/her early twenties. He had received a kidney transplant due to FSGS with ESRD 10 years earlier. In line with the initial diagnosis of FSGS an immunosuppressive therapy with prednisone, cyclosporine, and cyclophosphamide had been administered. One month following kidney transplantation, the patient had been successfully treated with cyclophosphamide and plasmapheresis due to relapsing FSGS [41]. 11 years after transplantation, laboratory testing showed a gradual loss of transplant function with elevated serum creatinine (3.3 mg/dl), prominent urinary protein (3.4 g/g), and hematuria. Transplant kidney biopsy revealed glomerular sclerosis, mesangial and endocapillary proliferation. Immunofluorescence and electron microscopy confirmed the diagnosis of de novo C3GN (Table 2; *C3GN4*, 1^st^ biopsy). Further analysis showed activation of the alternative pathway and a weakly positive C3NeF (Additional file 1: Table S6). Genetic testing revealed heterozygous changes of unknown clinical significance in *CFI* and *COL4A3* (Table 2, detailed discussion Additional file 1: Text S2).

**Fig. 2:**
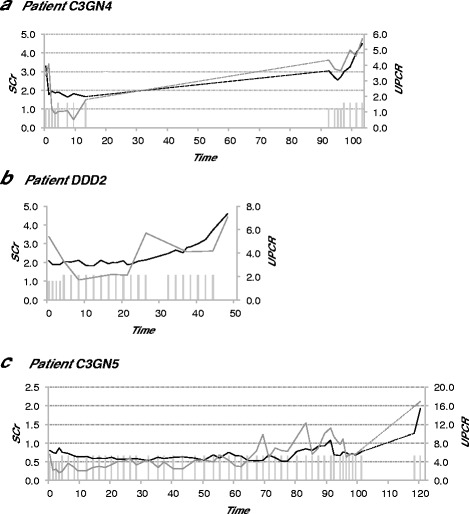
Negative response to eculizumab treatment in C3G patients. Graphs show treatment response to eculizumab. Column charts show applied eculizumab dose (low column: 900 mg; high column: 1200 mg). Line charts display serum serum creatinine levels (black; SCr; mg/dl), and urinary protein levels (grey; UPCR; g/g) over time (weeks). **a** Patient *C3GN4.* Dashed lines show the period of time (1.5 years) where eculizumab was paused. **b** Patient *DDD2*. **c** Patient *C3GN5.* Dashed lines show the period of time (17 weeks) where eculizumab was paused

Immunosuppression after kidney transplantation was maintained with prednisone, tacrolimus, and MMF. After administration of eculizumab, kidney transplant function and urinary protein greatly improved. Serum creatinine levels stabilized at 1.6–1.8 mg/dl and urinary protein levels at 1 g/g. However after 3 months of treatment, eculizumab was stopped due to lack of compliance. 1.5 years later the patient presented again with relapsing disease, serum creatinine levels of 3 mg/dl, and urinary protein of 4.3 g/g. A second kidney biopsy showed progressing glomerular sclerosis, and immunofluorescence revealed complement deposition but no IgG. (Table 2; *C3GN4*, 2^nd^ biopsy). Treatment with eculizumab was reapplied. Hematuria levels remained stable, however serum creatinine and urinary protein levels further increased, and treatment with eculizumab was discontinued after 3 months (Fig. 2a).

*Patient DDD2* presented with elevated serum creatinine levels (2.1 mg/dl), nephrotic range urinary protein (5.4 g/g), and hematuria at the in his/her late fifties (Additional file 1: Table S7). Kidney biopsy displayed mesangial proliferation, lymphocyte infiltration, and discrete glomerular sclerosis. Immunofluorescence showed distinct C3 staining with little immunoglobulin staining. Electron microscopy found dense deposits in accordance with DDD (Table 2). Further diagnostic workup revealed alternative pathway complement activation but no autoantibodies against C3NeF and CFH. Genetic analysis detected a heterozygous missense variant in *CFHR5* (Table 2, detailed discussion in Additional file 1: Text S3). Treatment with eculizumab was initiated stabilizing kidney function at serum creatinine levels of 2 mg/dl and urinary protein levels of 2 g/g. However, in the following months kidney function and urinary protein deteriorated. Thus, treatment with eculizumab was terminated and dialysis was initiated (Fig. 2b).

*Patient C3GN5* presented in his/her mid-teens with nephrotic range urinary protein (5.6 g/g), normal renal function (serum creatinine levels 0.8 mg/dl), and hematuria. Kidney biopsy showed pronounced glomerular sclerosis, mesangial proliferation, and distinct C3 staining with little immunoglobulin deposition. Electron microscopy confirmed the diagnosis of C3GN (Table 2, *C3GN5*, 1^st^ and 2^nd^ biopsy). Complement analysis revealed extensive activation of the alternative pathway with low C3 levels (Additional file 1: Table S8). Antibody and genetic testing detected C3NeF, but no mutation in known disease genes (Table 2). Treatment with plasmapheresis, plasma infusion, prednisone, and MMF had initially induced reduction of urinary protein (details published in [25]). Due to deterioration of urinary protein (5.6 g/g) eculizumab therapy was initiated. With the initiation of eculizumab, MMF was stopped but was readministered after 2.5 months since urinary protein increased. A follow-up biopsy after 1 year of eculizumab treatment revealed glomerular IgG, and κ light chain deposition with identical staining pattern for mouse antibodies (Table 2, *C3GN5*, 2^nd^ biopsy). Since eculizumab is a humanized monoclonal antibody with human IgG2/IgG4 heavy and κ light chain, and mouse variable regions [42], this glomerular antibody deposition may reflect eculizumab. A break of 17 weeks due to insurance payment negotiations led to a dramatic increase of urinary protein (> 15 g/g) and decline of renal function. Reinitiation of eculizumab could not halt disease progression (Fig. 2c).

## Discussion

We analyzed data of five patients with C3GN and two patients with DDD treated with eculizumab. The study population included patients with atypical history (patient *C3GN2* had a history of HIV infection, patient *C3GN4* presented with de novo C3GN in the kidney graft, and patient *DDD2* presented in his/her late fifties). We observed heterogeneous treatment response (Table 1): Four patients showed either improved or stable kidney function, and three patients did not respond. These latter cases also involved patient *C3GN4* who initially responded to eculizumab but showed disease progression in the second treatment attempt. Treatment response varied between 2 weeks and 6 months. This is in line with published literature as previous studies also reported varying response rates (Additional file 1: Table S1); [22, 26–39], although published literature shows a clear publication bias towards positive results. Our study supports that eculizumab can be a successful treatment option for at least some patients with C3G.

Although the numbers are small, this study indicates that timing and duration of treatment seem to be important factors for the effectiveness. As described in patient *C3GN2,* short treatment breaks may result in reversible loss of kidney function and urinary protein, while longer breaks (patients *C3GN4* and *C3GN5*) can result in disease progression, deterioration of kidney function, and treatment resistance. This is in line with recent publications showing that treatment brakes may result in relapsing disease [39, 43]. Our study also supports the notion that eculizumab is less likely to improve disease outcome in patients with more severe glomerular sclerosis and interstitial fibrosis. Especially patient *C3GN4*, who initially was successfully treated with eculizumab showed pronounced renal damage (> 50% glomerular sclerosis and IF/TA 25%) after a treatment break of 1.5 years, and resuming eculizumab treatment did not halt progression. In several studies, old age, renal impairment at presentation, and severe glomerular pathology were identified as predictors of ESRD in patients with C3G [6, 44]. While kidney biopsies provide important clues, systematic evaluation schemes are needed and correlation to treatment response and clinical outcome should be evaluated.

Several markers have been investigated that may help to evaluate efficacy of eculizumab. sMAC levels have been suggested as serum marker for the activity of the alternative complement pathway and treatment response to eculizumab [28]. However, several C3G cases have been reported with normal pre-treatment sMAC levels [5, 28, 36], and in several reports treatment response of C3G patients was heterogeneous without correlation to pre-treatment sMAC levels or the course of sMAC levels during eculizumab treatment [28, 29, 36]. In general, this questions sMAC levels as a treatment parameter. Further studies with detailed complement analyses are needed to guide therapeutic interventions.

Interestingly, we observed controlled hematuria in our collective. Two patients (*C3GN2* and *DDD1*) with decline in hematuria also paralleled improved kidney function suggesting reduced glomerular inflammation. A recent study also reported improved creatinine and partial reduction of hematuria in seven pediatric patients treated with eculizumab for DDD [36]. But, in two patients with deteriorating kidney function, hematuria partially receded (*DDD2* and *C3GN5)*. Additional research is needed to define clinical parameter for treatment response in C3G patients.

Of note, high range urinary protein might also lead to accelerated renal elimination of eculizumab and therefore weaken treatment response to eculizumab since therapeutic drug levels might not be maintained. In these cases, careful evaluation of complement activity such as CH50 as an indicator of total complement activity or eculizumab serum concentrations might be helpful in order to detect possible underdosing.

In contrast to aHUS, where eculizumab represents a successful therapy [40], response rates in C3G patients are heterogeneous. C3G and aHUS share uncontrolled activation of the alternative complement pathway and pathophysiological key regulators, such as mutations in complement factors or autoantibodies. However, the two diseases display fundamental differences: In aHUS, complement activation occurs on endothelial surfaces, leading to endothelial damage and thrombus formation. In C3G, complement activation occurs predominantly in the fluid phase resulting in excess formation of activated C3 and cleavage products, which are deposited in the kidney and raise glomerular damage [44]. Despite eculizumab-driven blockage of the terminal complement cascade, upstream C3 cleavage products may cause continuing glomerular damage. This provides reasons for diverging response rates to eculizumab in aHUS and C3G. Therefore, further studies evaluating other complement inhibitors such as the emerging group of C3-convertase inhibitors are needed. In this context, genetic and immunologic screening with registration of relevant mutations, such as the *CFHR5* genotype of patient *DDD2* in C3G databases, is crucial for understanding the pathophysiology and correlating genetic features with treatment response.

Since treatment with eculizumab in C3G patients as well as in aHUS likely needs to be maintained lifelong, long-term effects such as glomerular eculizumab deposition as seen in patient *C3GN5* and a previous study [45] have to be monitored. In this regard, the follow-up periods of this study as well as published literature (Additional file 1: Table S1) are too short to draw conclusions.

The time to allograft recurrence in the two reported patients varies widely (in patient *C3GN1* disease repalsed after 3 years, and in patient *C3GN3* after 3 months), which is in line with previous reports [12]. Rituximab has been discussed as a treatment option in autoantibody positive transplant recurrent C3G [46]. In our study (patient *C3GN1*), it did not affect disease progression and a previous report showed poor outcome [12]. However, reported numbers are too small to draw conclusions. As reported in this and previous studies (Additional file 1: Table S1), allograft recurrent C3GN can be treated with eculizumab. As recurrence rates are high, close clinical monitoring is especially important in this group.

This study has several limitations, the major being the retrospective character and the lack of standardized follow-up, including genetic and serologic testing. Also the multitude of therapeutic approaches prior to therapy with eculizumab both in this study and published literature (Additional file 1: Table S1) complicate the interpretation of the clinical course. This is mainly due to the rarity of C3 glomerulopathy, and consequently underlines the need to prospectively collect data of patients with C3G.

## Conclusions

Together, our series and previous case reports suggest that early diagnosis and continuous treatment might be necessary to affect the outcome of C3G. Treatment with eculizumab may be successful in some patients. Prospective long-term studies will be required to identify parameters that predict response to therapy.

## Additional file

Additional file 1: Text S1. Summary of genetic testing of patient *C3GN3.* **Text S2.** Summary of genetic testing of patient *C3GN4.* **Text S3.** Summary of genetic testing of patient *DDD2*. **Table S1.** Details on published cases with patient characteristics, and treatment response. **Table S2.** Details on treatment and response of patient *C3GN1*. **Table S3.** Details on treatment and response of patient *C3GN2*. **Table S4.** Details on treatment and response of patient *C3GN3*. **Table S5.** Details on treatment and response of patient DDD1. **Table S6.** Details on treatment and response of patient *C3GN4*. **Table S7.** Details on treatment and response of patient *DDD2*. **Table S8.** Details on treatment and response of patient *C3GN5. (DOCX 155 kb)*

## Abbreviations

ACEI: Angiotensin converting enzyme inhibitors
aHUS: atypical hemolytic uremic syndrome
ARB: Angiotensin receptor blockers
C3G: C3 glomerulopathy
C3GN: C3 glomerulonephritis
C3NeF: C3 nephritic factor
CyA: Cyclosporine A
DDD: Dense deposit disease
DEAP-HUS: Deficiency of complement factor H-related plasma proteins and autoantibody positive form of hemolytic uremic syndrome
Del: Deletion
eGFR: estimated glomerular filtration rate
ESRD: End-stage renal disease
FFP: Fresh frozen plasma
HET: Heterozygous
HIV: Human immunodeficiency virus
HOM: Homozygous
IF/TA: Interstitial fibrosis and tubular atrophy
MPGN: Membranoproliferative glomerulonephritis
NT: Not tested
PEX: Plasmapheresis
SCr: Serum creatinine
sMAC: soluble membrane attack complex
UPCR: Urinary protein to serum protein ratio

## References

[1] Sethi S, Fervenza FC (2012). Membranoproliferative glomerulonephritis--a new look at an old entity. N Engl J Med.

[2] Sethi S, Nester CM, Smith RJ (2012). Membranoproliferative glomerulonephritis and C3 glomerulopathy: resolving the confusion. Kidney Int.

[3] Bomback AS, Appel GB (2012). Pathogenesis of the C3 glomerulopathies and reclassification of MPGN. Nat Rev Nephrol.

[4] Pickering MC, D'Agati VD, Nester CM, Smith RJ, Haas M, Appel GB, Alpers CE, Bajema IM, Bedrosian C, Braun M (2013). C3 glomerulopathy: consensus report. Kidney Int.

[5] Iatropoulos P, Noris M, Mele C, Piras R, Valoti E, Bresin E, Curreri M, Mondo E, Zito A, Gamba S (2016). Complement gene variants determine the risk of immunoglobulin-associated MPGN and C3 glomerulopathy and predict long-term renal outcome. Mol Immunol.

[6] Medjeral-Thomas NR, O'Shaughnessy MM, O'Regan JA, Traynor C, Flanagan M, Wong L, Teoh CW, Awan A, Waldron M, Cairns T (2014). C3 glomerulopathy: clinicopathologic features and predictors of outcome. Clin J Am Soc Nephrol.

[7] Marks SD, Rees L (2000). Spontaneous clinical improvement in dense deposit disease. Pediatr Nephrol.

[8] Lu DF, Moon M, Lanning LD, McCarthy AM, Smith RJ (2012). Clinical features and outcomes of 98 children and adults with dense deposit disease. Pediatr Nephrol.

[9] Nasr SH, Valeri AM, Appel GB, Sherwinter J, Stokes MB, Said SM, Markowitz GS, D'Agati VD (2009). Dense deposit disease: clinicopathologic study of 32 pediatric and adult patients. Clin J Am Soc Nephrol.

[10] Servais A, Noel LH, Roumenina LT, Le Quintrec M, Ngo S, Dragon-Durey MA, Macher MA, Zuber J, Karras A, Provot F (2012). Acquired and genetic complement abnormalities play a critical role in dense deposit disease and other C3 glomerulopathies. Kidney Int.

[11] Alasfar S, Carter-Monroe N, Rosenberg AZ, Montgomery RA, Alachkar N (2016). Membranoproliferative glomerulonephritis recurrence after kidney transplantation: using the new classification. BMC Nephrol.

[12] Zand L, Lorenz EC, Cosio FG, Fervenza FC, Nasr SH, Gandhi MJ, Smith RJ, Sethi S (2014). Clinical findings, pathology, and outcomes of C3GN after kidney transplantation. J Am Soc Nephrol.

[13] Barbour TD, Pickering MC, Cook HT (2013). Recent insights into C3 glomerulopathy. Nephrol Dial Transplant.

[14] Sethi S, Fervenza FC (2014). Pathology of renal diseases associated with dysfunction of the alternative pathway of complement: C3 glomerulopathy and atypical hemolytic uremic syndrome (aHUS). Semin Thromb Hemost.

[15] Pickering M, Cook HT (2011). Complement and glomerular disease: new insights. Curr Opin Nephrol Hypertens.

[16] Sethi S, Fervenza FC, Zhang Y, Nasr SH, Leung N, Vrana J, Cramer C, Nester CM, Smith RJ (2011). Proliferative glomerulonephritis secondary to dysfunction of the alternative pathway of complement. Clin J Am Soc Nephrol.

[17] Roumenina LT, Loirat C, Dragon-Durey MA, Halbwachs-Mecarelli L, Sautes-Fridman C, Fremeaux-Bacchi V (2011). Alternative complement pathway assessment in patients with atypical HUS. J Immunol Methods.

[18] Smith RJ, Harris CL, Pickering MC (2011). Dense deposit disease. Mol Immunol.

[19] Goodship TH, Cook HT, Fakhouri F, Fervenza FC, Fremeaux-Bacchi V, Kavanagh D, Nester CM, Noris M, Pickering MC, Rodriguez de Cordoba S (2017). Atypical hemolytic uremic syndrome and C3 glomerulopathy: conclusions from a “kidney disease: improving global outcomes” (KDIGO) controversies conference. Kidney Int.

[20] Appel GB, Cook HT, Hageman G, Jennette JC, Kashgarian M, Kirschfink M, Lambris JD, Lanning L, Lutz HU, Meri S (2005). Membranoproliferative glomerulonephritis type II (dense deposit disease): an update. J Am Soc Nephrol.

[21] Giaime P, Daniel L, Burtey S (2015). Remission of C3 glomerulopathy with rituximab as only immunosuppressive therapy. Clin Nephrol.

[22] McCaughan JA, O'Rourke DM, Courtney AE (2012). Recurrent dense deposit disease after renal transplantation: an emerging role for complementary therapies. Am J Transplant.

[23] Rabasco C, Cavero T, Roman E, Rojas-Rivera J, Olea T, Espinosa M, Cabello V, Fernandez-Juarez G, Gonzalez F, Avila A (2015). Effectiveness of mycophenolate mofetil in C3 glomerulonephritis. Kidney Int.

[24] Licht C, Heinen S, Jozsi M, Loschmann I, Saunders RE, Perkins SJ, Waldherr R, Skerka C, Kirschfink M, Hoppe B (2006). Deletion of Lys224 in regulatory domain 4 of factor H reveals a novel pathomechanism for dense deposit disease (MPGN II). Kidney Int.

[25] Haffner K, Michelfelder S, Pohl M (2015). Successful therapy of C3Nef-positive C3 glomerulopathy with plasma therapy and immunosuppression. Pediatr Nephrol.

[26] Daina E, Noris M, Remuzzi G (2012). Eculizumab in a patient with dense-deposit disease. N Engl J Med.

[27] Vivarelli M, Pasini A, Emma F (2012). Eculizumab for the treatment of dense-deposit disease. N Engl J Med.

[28] Bomback AS, Smith RJ, Barile GR, Zhang Y, Heher EC, Herlitz L, Stokes MB, Markowitz GS, D'Agati VD, Canetta PA (2012). Eculizumab for dense deposit disease and C3 glomerulonephritis. Clin J Am Soc Nephrol.

[29] Gurkan S, Fyfe B, Weiss L, Xiao X, Zhang Y, Smith RJ (2013). Eculizumab and recurrent C3 glomerulonephritis. Pediatr Nephrol.

[30] Besbas N, Gulhan B, Gucer S, Korkmaz E, Ozaltin F (2014). A novel CFHR5 mutation associated with C3 glomerulonephritis in a Turkish girl. J Nephrol.

[31] Rousset-Rouviere C, Cailliez M, Garaix F, Bruno D, Laurent D, Tsimaratos M (2014). Rituximab fails where eculizumab restores renal function in C3nef-related DDD. Pediatr Nephrol.

[32] Kerns E, Rozansky D, Troxell ML (2013). Evolution of immunoglobulin deposition in C3-dominant membranoproliferative glomerulopathy. Pediatr Nephrol.

[33] Ozkaya O, Nalcacioglu H, Tekcan D, Genc G, Meydan BC, Ozdemir BH, Baysal MK, Keceligil HT (2014). Eculizumab therapy in a patient with dense-deposit disease associated with partial lipodystropy. Pediatr Nephrol.

[34] Berthe-Aucejo A, Sacquepee M, Fila M, Peuchmaur M, Perrier-Cornet E, Fremeaux-Bacchi V, Deschenes G (2014). Blockade of alternative complement pathway in dense deposit disease. Case Rep Nephrol.

[35] Sanchez-Moreno A, De la Cerda F, Cabrera R, Fijo J, Lopez-Trascasa M, Bedoya R, Rodriguez de Cordoba S, Ybot-Gonzalez P (2014). Eculizumab in dense-deposit disease after renal transplantation. Pediatr Nephrol.

[36] Oosterveld MJ, Garrelfs MR, Hoppe B, Florquin S, Roelofs JJ, van den Heuvel LP, Amann K, Davin JC, Bouts AH, Schriemer PJ (2015). Eculizumab in pediatric dense deposit disease. Clin J Am Soc Nephrol.

[37] Le Quintrec M, Lionet A, Kandel C, Bourdon F, Gnemmi V, Colombat M, Goujon JM, Fremeaux-Bacchi V, Fakhouri F (2015). Eculizumab for treatment of rapidly progressive C3 glomerulopathy. Am J Kidney Dis.

[38] Tran CL, Sethi S, Murray D, Cramer CH, Sas DJ, Willrich M, Smith RJ, Fervenza FC (2016). Discontinuation of dialysis with eculizumab therapy in a pediatric patient with dense deposit disease. Pediatr Nephrol.

[39] Lebreton C, Bacchetta J, Dijoud F, Bessenay L, Fremeaux-Bacchi V, Sellier-Leclerc AL (2017). C3 glomerulopathy and eculizumab: a report on four paediatric cases. Pediatr Nephrol.

[40] Legendre CM, Licht C, Muus P, Greenbaum LA, Babu S, Bedrosian C, Bingham C, Cohen DJ, Delmas Y, Douglas K (2013). Terminal complement inhibitor eculizumab in atypical hemolytic-uremic syndrome. N Engl J Med.

[41] Haffner K, Zimmerhackl LB, von Schnakenburg C, Brandis M, Pohl M (2005). Complete remission of post-transplant FSGS recurrence by long-term plasmapheresis. Pediatr Nephrol.

[42] Rother RP, Rollins SA, Mojcik CF, Brodsky RA, Bell L (2007). Discovery and development of the complement inhibitor eculizumab for the treatment of paroxysmal nocturnal hemoglobinuria. Nat Biotechnol.

[43] Payette A, Patey N, Dragon-Durey MA, Fremeaux-Bacchi V, Le Deist F, Lapeyraque AL (2015). A case of C3 glomerulonephritis successfully treated with eculizumab. Pediatr Nephrol.

[44] Ramadass M, Ghebrehiwet B, Smith RJ, Kew RR (2014). Generation of multiple fluid-phase C3b:plasma protein complexes during complement activation: possible implications in C3 glomerulopathies. J Immunol.

[45] Herlitz LC, Bomback AS, Markowitz GS, Stokes MB, Smith RN, Colvin RB, Appel GB, D'Agati VD (2012). Pathology after eculizumab in dense deposit disease and C3 GN. J Am Soc Nephrol.

[46] Thomas S, Ranganathan D, Francis L, Madhan K, John GT (2014). Current concepts in C3 glomerulopathy. Indian J Nephrol.

