# Supplementary material for: Treating C3 glomerulopathy with eculizumab
Source: BMC Nephrol. 2018 Jan 12;19:7. doi: 10.1186/s12882-017-0802-4 (PMC5767001; doi:10.1186/s12882-017-0802-4)
Supplement: Additional file 1: Text S1. — Summary of genetic testing of patient C3GN3. Text S2. Summary of genetic testing of patient C3GN4. Text S3. Summary of genetic testing of patient DDD2. Table S1. Details on published cases with patient characteristics, and treatment response. Table S2. Details on treatment and response of patient C3GN1. Table S3. Details on treatment and response of patient C3GN2. Table S4. Details on treatment and response of patient C3GN3. Table S5. Details on treatment and response of patient DDD1. Table S6. Details on treatment and response of patient C3GN4. Table S7. Details on treatment and response of patient DDD2. Table S8. Details on treatment and response of patient C3GN5. (DOCX 155 kb) [file 12882_2017_802_MOESM1_ESM.docx]

**Supplementary Material to**

**Treating C3 Glomerulopathy with Eculizumab**

Thomas Welte, MD; Frederic Arnold, MD; Julia Kappes, MD; Maximilian Seidl, MD; Karsten Häffner, MD; Carsten Bergmann, MD; Gerd Walz, MD; Elke Neumann-Haefelin, MD ^1^

^1^ Corresponding author.

Elke Neumann-Haefelin, MD

Department of Nephrology, Medical Center – University of Freiburg, Germany

Hugstetter Strasse 55, 79106 Freiburg, Germany

E-mail: elke.neumann-haefelin@uniklinik-freiburg.de

Phone: +49 761 270 35590

Fax: +49 761 270 32700

**This PDF File includes:**

Supplementary Texts 1 – 3

Supplementary Tables 1 – 8

References for Supplementary Material

**Supplementary Texts**

**Additional Text S1. Genetic testing of patient *C3GN3***

A heterozygous nucleotide exchange in exon 17 of *C3* (c.2203C<T) was detected leading to an amino acid change in the protein (R725W). This mutation was previously associated with aHUS [1]. However, *in vitro* studies revealed normal ligand binding and protein activity [1] questioning the pathophysiological relevance of the mutation.

**Additional Text S2. Genetic testing of patient *C3GN4***

In the *CFI* gene, a heterozygous mutation was detected (c.1322A>G) leading to an amino acid change (K441R) in the protein. Although this mutation has been described in a patient with aHUS [2], five *in silico* tools [3-7] predicted no impaired protein function, making the pathophysiological relevance of this mutation questionable. In addition, a heterozygous *CFHR1-3* deletion was detected. While homozygous *CFHR1-3* deletions have been described in patients with DEAP-HUS [8, 9], heterozygous deletions have been associated with aHUS, but are also found in a considerable proportion of healthy individuals [10, 11]. In the *COL4A3* gene, a heterozygous variant (c.4484A>G) was detected resulting in an amino acid exchange in the protein (Q1495R). Three bioinformatic tools predicted impaired protein function [3, 4, 6], whereas two were in favor of normal protein function [5, 7]. Segregation analysis showed the mutation to be inherited maternally. As the mother was healthy, the relevance of the mutation is questionable.

**Additional Text S3. Genetic testing of patient *DDD2***

Genetic testing revealed a pathophysiologically questionable heterozygous missense variant in *cfhr5* (c.832G>A) leading to an amino acid exchange (G278R) in the protein, which has not been described previously. Three bioinformatic tools predicted impaired protein function [4, 5, 7].

**Additional file Tables**

**Additional file: Table S1. Literature review: Published cases, patient characteristics, and treatment response**

| **Ref.** | **Diagnosis (age/sex)** | **native/graft^a^** | **Baseline SCr (mg/dl)** | **Baseline UPCR (g/g)** | **Previous therapy** | **Notable antibody/**  **genetic testing** | **Time diagnosis to Eculizumab (months)** | **Treatment duration (months)** | **Response SCr^b^** | **Response UPCR^b^** |
| --- | --- | --- | --- | --- | --- | --- | --- | --- | --- | --- |
| [12] | **DDD**  **(22M)** | native | 2.0 | 0.7 | none | *CFH* c.2867C>T | 25 | 12 | **yes** | **stable** |
|  | **DDD**  **(42M)** | native | 1.2 | 4.5 | steroid (native); tacrolimus, PEX (graft) | none | 150 (native); 0.5 (graft) | 12 | **no** | **yes** |
|  | **DDD**  **(32M)** | native | 1.9 | 3.5 | steroid | C3Nef | 332 | 9 | **no** | **no** |
|  | **C3GN (20M)** | native | 1.7 | 0.1 | tacrolimus, MMF, rituximab (native); steroid, tacrolimus, MMF (graft) | C3Nef;  *MCP* c.475+1G>A | 114 (native); 2 (graft) | 12 | **yes** | **stable** |
|  | **C3GN (25M)** | native | 1.6 | 2.6 | steroid, MMF | none | 162 | 12 | **no** | **yes** |
|  | **C3GN (22M)** | native | 1.8 | 4.4 | steroid (native); steroid, tacrolimus, MMF (graft) | C3Nef | 138 (native); 8 (graft) | 12 | **no** | **stable** |
| [13] | **DDD**  **(63F)** | graft | 2.2 | 0.16 | steroid, PEX | none | 5 | 32 | **yes** | **yes** |
|  | **C3GN (27F)** | native | 6 | 1.42 | none | none | 8 | 19 | **yes** | **yes** |
|  | **C3GN (45M)** | native | 4.1 | 1.3 | steroid, MMF | none | 15 | 6 | **yes** | **yes** |
| [14] | **DDD**  **(11F)** | native | 2.2 | 5.4 | steroid, rituximab | C3Nef;  *CFH* HOM I62V and H402Y | not specified | 11 | **yes** | **yes** |
| [15] | **DDD**  **(10M)** | native | 1.1 | 3.5 to 5.5 | none | C3Nef;  *CFB* polymor-phisms C94T/R32W; G95A/R32Q | not specified | 18; 9 | **stable; stable** | **yes;**  **yes** |
| [16] | **DDD**  **(7 native; 29 graft F)** | graft | 4.9 | 7.6 | steroid, rituximab, PEX | none | not specified (native);  2 (graft) | 3 | **yes** | **yes** |
| [17] | **C3GN**  **(8 native; 19 graft M)** | graft | 1.5 | not specified | rituximab | C3Nef,  *CFH* polymor-phisms rs800292, rs1061170*, C3* polymor-phisms rs2330199, rs1047286 | not specified (native);  3 (graft) | 12 | **yes** | **yes** |
| [18] | **C3GN (13M)** | native | 0.9 | 9.8 | steroid, MMF, rituximab | none | 44 | not specified | **stable** | **yes** |
| [19] | **DDD**  **(8M)** | native | not specified | not specified | steroid, MMF rituximab | C3Nef | 14 | 6.5 | **yes** | **yes** |
| [20] | **DDD**  **(14F)** | native | 0.5 | 10 | steroid, cyclophos-phamide, dipyridamole, PEX | C3Nef,  *CFH* polymor-phisms V62I, H402Y | not specified | 7 | **stable** | **yes** |
| [21] | **DDD**  **(15M)** | native | not specified | 2.6 | steroid, PEX, rituximab | C3Nef,  *CFH* R232X | not specified | 3.6 | **stable** | **stable** |
| [22] | **DDD**  **(8 native; 14 graft F)** | graft | 1,1 | unclear | steroid, PEX, rituximab (native); basiliximab, steroid, tacrolimus, MMF, PEX (graft) | C3Nef | not specified (native);  not specified (graft) | 30 | **yes** | **stable** |
| [23] | **DDD**  **(13F)** | native | hemo-dialysis | 12.8 | steroid, cyclophos-phamide, PEX | *CFH*  HOM V62 + H402;  *C3* HOM G102 + L314;  *ADAMTS13* HET p.C1275X | not specified | 8 | **yes** | **yes** |
| [24] | **DDD**  **(13F)** | native | 1.4 | 17 | steroid, PEX | *CFHR1-3* HOM del | 0.9 | 3 | **yes** | **yes** |
|  | **DDD**  **(6M)** | native | 2.5;  0.6 | 14.8;  8.5 | steroid, PEX;  none | C3Nef; *CFHR1-3* HET del | 0.6;  0.1 | 3;  3 | **yes;**  **stable** | **yes;**  **yes** |
|  | **DDD**  **(7F)** | native | 1.1 | 9.6 | none | C3Nef | 18.2 | 3 | **yes** | **yes** |
|  | **DDD**  **(6F)** | native | 3.9;  1.7 | 7.7;  6.8 | steroid, PEX; steroid | C3Nef;  *CFHR1-3* HET del | 0.2;  7.8 | 4.5;  3 | **yes;**  **yes** | **yes;**  **yes** |
|  | **DDD**  **(2M)** | native | 0.8 | 2.2 | cyclo-sporine A | C3Nef | 119 | 3 | **stable** | **yes** |
| [25] | **C3G**  **(9M)** | native | no renal failure; not specified | not specified | none | C3Nef | not specified | 19 | **stable** | **yes** |
|  | **C3G**  **(13F)** | native | no renal failure; not specified | not specified | steroid, MMF | C3Nef | not specified | 19 | **stable** | **yes** |
|  | **C3G**  **(12M)** | native | 2.0 | not specified | steroid, MMF | C3Nef | not specified | 7 | **yes** | **yes** |
|  | **C3G**  **(9F)** | graft | renal failure; not specified | not specified | thymo-globulins, steroids, MMF, tacrolimus, PEX, immuno-globulins | none | not specified (native);  not specified (graft) | 6 | **not specified** | **no** |
| [26] | **C3GN**  **(5M)** | native | 0.2 | 4.7 | steroid, rituximab, MMF | C3NeF, CFH, *CFI* HET I398L | 36 | 19 relapsing disease (eculizu-mab paused 2 months); 15 | **stable** | **yes** |
| [27] | **C3GN**  **(38F)** | native | 11 | 6.8 | steroid, PEX | *CFHR3/1* HOM del polymor-phism | not specified | not specified | **yes** | **yes** |
| [28] | **C3GN (16F)** | native | 0.5 | 2.0 | steroid | *CFHR5* HOM c.805T>C; *CFI* HOM polymor-phism rs11098044 | 3 | 10 | **stable** | **no** |

^a^ native: applies to native kidney; graft: applies to kidney transplant; ^b^ positive, stable or negative response has been adopted according to statements from corresponding publications. Abbreviations: del., deletion; HET, heterozygous; HOM, homozygous; MMF, mycophenolate mofetil; PEX, plasmapheresis.

**Additional file Table S2. Treatment and response patient *C3GN1***

| **Week** | **Eculizu-**  **mab (mg)** | **BUN (mg/dl)** | **SCr**  **(mg/dl)** | **eGFR (ml/min/**  **1.73m^2^)** | **TP^a^ (g/dl)** | **SA^b^ (g/dl)** | **UPCR (g/g)** | **Hematuria^c^ (0-4)** | **C3^d^ (g/l)** | **C3d^e^ (mg/l)** | **C4^f^ (g/l)** | **CH50^g^ (E/ml)** |
| --- | --- | --- | --- | --- | --- | --- | --- | --- | --- | --- | --- | --- |
| **-14** |  |  |  |  |  |  |  |  | 0,4 | 8,0 | 0,1 | 18 |
| **0** | 900 | 70 | 1.9 | 42.4 |  |  |  |  |  |  |  |  |
| **1** | 900 | 61 | 1.6 | 50.5 |  | 1.9 | 5.4 | 4 |  |  |  |  |
| **2** | 900 | 47 | 1.4 | 58.7 |  |  | 6.1 | 4 |  |  |  |  |
| **3** | 900 | 50 | 1.3 | 64.9 |  |  |  |  |  |  |  |  |
| **4** | 1200 | 47 | 1.4 | 60.6 |  |  |  | 4 |  |  |  |  |
| **7** | 1200 | 59 | 1.6 | 53.1 |  | 2.6 | 3.9 | 4 | 0.4 | 8.0 |  | 18 |
| **9** | 1200 | 65 | 1.8 | 45.9 |  | 3.2 | 2.8 | 4 |  |  |  |  |
| **11** | 1200 | 69 | 1.8 | 46.2 | 8.0 | 3.8 | 2.7 | 4 |  |  |  |  |
| **13** | 1200 | 80 | 1.9 | 41.8 |  | 4.0 | 2.5 | 4 |  |  |  |  |
| **15** | 1200 | 80 | 2.0 | 39.9 |  |  | 1.9 | 4 |  |  |  |  |
| **17** | 1200 | 77 | 1.8 | 45.1 |  |  | 1.6 | 4 | 0.3 | 17.4 | 0.1 | > 10 |
| **19** | 1200 | 84 | 1.9 | 43.7 |  | 4.3 | 0.9 | 4 |  |  |  |  |
| **21** | 1200 | 96 | 1.9 | 42.6 |  |  | 0.8 | 4 |  |  |  |  |
| **23** | 1200 | 112 | 1.9 | 42.9 |  | 4.5 | 0.5 | 4 |  |  |  |  |
| **25** | 1200 | 70 | 1.7 | 47.2 |  | 4.1 | 0.7 | 4 |  |  |  |  |
| **27** | 1200 | 91 | 1.9 | 43.4 |  |  | 0.4 |  |  |  |  |  |
| **29** | 1200 | 104 | 1.8 | 44.2 |  |  | 0.4 | 4 |  |  |  |  |
| **31** | 1200 | 76 | 2.0 | 41.1 |  |  | 0.4 | 4 |  |  |  |  |
| **33** | 1200 | 79 | 1.8 | 45.1 |  |  | 0.4 | 4 |  |  |  |  |
| **35** | 1200 | 84 | 1.7 | 47.5 |  |  | 0.4 | 4 |  |  |  |  |
| **37** | 1200 | 82 | 1.9 | 43.9 |  | 4.5 | 0.5 | 4 |  |  |  |  |
| **39** | 1200 | 91 | 2.0 | 40.9 |  |  | 0.3 | 3 |  |  |  |  |
| **41** | 1200 | 71 | 1.7 | 47.8 |  |  |  |  |  |  |  |  |
| **43** | 1200 | 68 | 1.9 | 43.7 |  |  | 0.4 | 3 | 0.5 | 20.7 | 0.1 | < 10 |
| **45** | 1200 | 60 | 1.7 | 50.1 |  |  |  |  |  |  |  |  |
| **47** | 1200 | 94 | 2.1 | 38.8 |  |  | 0.3 | 4 |  |  |  |  |
| **49** | 1200 | 65 | 1.8 | 44.5 |  |  |  |  |  |  |  |  |
| **51** | 1200 | 88 | 2.1 | 38.8 |  |  | 0.2 | 3 | 0.4 | 16.1 | 0.1 |  |
| **53** | 1200 | 98 | 1.8 | 44.5 |  |  |  |  |  |  |  |  |
| **55** | 1200 | 61 | 1.9 | 43.4 |  |  | 0.3 | 3 |  |  |  |  |
| **57** | 1200 | 63 | 1.8 | 44.0 |  |  |  |  |  |  |  |  |
| **59** | 1200 | 68 | 2.2 | 36.0 |  | 4.6 | 0.3 | 3 |  |  |  |  |
| **61** | 1200 | 83 | 1.9 | 42.9 |  |  |  |  |  |  |  |  |
| **63** | 1200 | 35 | 2.2 | 35.0 |  |  | 0.2 | 3 |  |  |  |  |
| **65** | 1200 | 89 | 2.1 | 38.2 |  |  |  |  |  |  |  |  |
| **67** | 1200 | 80 | 1.9 | 42.4 |  |  | 0.3 | 3 |  |  |  |  |
| **69** | 1200 | 80 | 2.1 | 36.9 |  |  |  |  |  |  |  |  |
| **71** | 1200 | 85 | 2.1 | 37.1 |  |  | 0.2 | 2 |  |  |  |  |
| **73** | 1200 | 65 | 1.7 | 47.2 |  |  |  |  |  |  |  |  |
| **75** | 1200 | 63 | 1.9 | 43.2 |  |  | 0.2 | 1 |  |  |  |  |
| **77** | 1200 | 92 | 1.9 | 42.4 |  |  |  |  |  |  |  |  |
| **79** | 1200 | 69 | 1.8 | 44.8 |  |  | 0.4 | 3 |  |  |  |  |
| **81** | 1200 | 72 | 1.9 | 43.4 |  |  |  |  |  |  |  |  |
| **83** | 1200 | 72 | 1.7 | 47.5 |  |  |  |  |  |  |  |  |
| **84** | 1200 | 64 | 1.6 | 50.6 |  |  | 0.4 | 2 |  |  |  |  |
| **86** | 1200 | 70 | 1.7 | 47.2 |  |  |  |  |  |  |  |  |
| **88** | 1200 | 67 | 1.8 | 46.0 |  |  | 0.2 | 2 |  |  |  |  |
| **90** | 1200 | 68 | 1.7 | 48.2 |  |  |  |  |  |  |  |  |
| **92** | 1200 | 69 | 1.7 | 47.5 |  |  | 0.4 | 2 |  |  |  |  |
| **94** | 1200 | 61 | 1.7 | 47.9 |  |  |  |  |  |  |  |  |
| **96** | 1200 | 64 | 1.8 | 46.6 |  |  | 0.3 | 2 |  |  |  |  |
| **98** | 1200 | 69 | 1.7 | 48.5 |  |  |  |  |  |  |  |  |
| **100** | 1200 | 74 | 1.9 | 42.4 |  |  | 0.3 | 2 |  |  |  |  |
| **102** | 1200 | 67 | 1.7 | 47.9 |  |  |  |  |  |  |  |  |
| **104** | 1200 | 64 | 1.7 | 49.9 |  |  | 0.3 | 3 |  |  |  |  |
| **106** | 1200 | 56 | 1.6 | 50.6 |  |  |  |  |  |  |  |  |
| **108** | 1200 | 68 | 1.7 | 47.2 |  |  | 0.2 | 3 |  |  |  |  |
| **110** | 1200 | 79 | 1.7 | 47.9 |  |  |  |  |  |  |  |  |
| **112** | 1200 | 68 | 1.6 | 52.0 |  |  | 0.2 | 3 |  |  |  |  |
| **114** | 1200 | 62 | 1.6 | 51.7 |  |  |  |  |  |  |  |  |
| **116** | 1200 | 60 | 1.6 | 50.9 |  |  | 0.5 | 3 |  |  |  |  |

^a^ TP, total serum protein; ^b^ SA, serum albumin; ^c^ hematuria measured by urine dipstic (scale from 0 to 4); ^d^ complement C3 ref. range: 0.9 – 1.8 g/l; ^e^ complement C3d ref. range: < 9 mg/l; ^f^ complement C4 ref. range: 0.1 – 0.4 g/l; ^g^ complement CH50 ref. range: 20 – 50 E/ml.

**Additional file Table S3. Treatment and response patient *C3GN2***

| **Week** | **Eculizu-**  **mab**  **(mg)** | **BUN**  **(mg/dl)** | **SCr**  **(mg/dl)** | **eGFR**  **(ml/min/**  **1.73m^2^)** | **TP^a^**  **(g/dl)** | **SA^b^**  **(g/dl)** | **UPCR**  **(g/g)** | **Hematuria^c^**  **(0-4)** | **C3^d^**  **(g/l)** | **C3d^e^**  **(mg/l)** | **C4^f^**  **(g/l)** | **CH50^g^**  **(E/ml)** | **sMAC^h^**  **(ng/ml)** |
| --- | --- | --- | --- | --- | --- | --- | --- | --- | --- | --- | --- | --- | --- |
| **-4** |  |  |  |  |  |  |  |  |  |  |  |  | 994 |
| **0** | 900 | 166 | 2.3 | 25.3 |  |  | 0.3 | 4 | 0.7 | 62.3 | 0.1 | < 10 |  |
| **1** | 900 | 95 | 1.9 | 31.9 |  |  |  |  |  |  |  |  |  |
| **2** | 900 | 71 |  |  |  |  |  |  |  |  |  |  |  |
| **3** | 900 | 72 | 1.5 | 42.0 |  |  |  |  |  |  |  |  |  |
| **5** | 1200 | 74 | 1.3 | 46.7 |  |  | 0.2 | 4 | 0.4 | 84,3 | 0.1 | < 10 |  |
| **7** | 1200 | 51 | 1.5 | 51.1 |  |  |  |  |  |  |  |  |  |
| **10** | 1200 | 70 | 1.4 | 41.6 |  |  |  |  |  |  |  |  |  |
| **12** | 1200 | 69 | 1.5 | 43.7 |  |  |  |  |  |  |  |  |  |
| **13** |  |  |  |  |  |  | 0.4 | 4 | 0.4 | 72.5 | 0.0 | < 10 | 247 |
| **14** | 1200 | 92 | 1.5 | 42.6 |  |  |  | 4 |  |  |  |  |  |
| **20** | 1200 | 89 | 1.3 | 39.5 |  |  |  |  |  |  |  |  |  |
| **22** | 1200 | 65 | 1.8 | 39.5 |  |  |  |  |  |  |  |  |  |
| **24** | 1200 |  |  |  |  |  |  |  |  |  |  |  |  |
| **25** |  | 46 | 1.8 | 39.5 |  |  |  | 4 |  |  |  |  |  |
| **26** | 1200 |  |  |  |  |  |  |  |  |  |  |  |  |
| **27** |  |  | 1.9 | 31.4 |  |  | 0.3 | 4 |  |  |  |  |  |
| **29** | 1200 | 60 | 1.1 | 52.1 |  |  |  |  |  |  |  |  |  |
| **31** | 1200 | 50 | 1.3 | 49.7 |  |  |  |  |  |  |  |  |  |
| **33** | 1200 |  | 1.3 | 49.7 |  |  |  |  |  |  |  |  |  |
| **34** |  |  |  |  |  |  | 0.2 | 4 |  |  |  |  |  |
| **35** | 1200 | 61 | 1.7 | 35.2 |  |  |  | 4 |  |  |  |  |  |
| **39** | 1200 | 52 | 1.6 | 38.1 |  |  |  |  |  |  |  |  |  |
| **40** |  | 60 | 1.7 | 35.2 |  |  |  |  |  |  |  |  |  |
| **42** |  |  | 2.2 |  |  |  | 1.3 | 4 |  |  |  |  | 914 |
| **51** | 1200 | 53 | 1.8 | 33.7 |  |  |  |  |  |  |  |  |  |
| **52** |  |  |  |  |  |  | 1.8 | 4 |  |  |  |  |  |
| **54** | 1200 | 69 | 1.8 | 33.7 |  |  |  |  |  |  |  |  |  |
| **57** | 1200 | 62 | 1.9 | 31.4 |  |  |  |  |  |  |  |  |  |
| **59** | 1200 |  |  |  |  |  |  |  |  |  |  |  |  |
| **60** |  | 69 | 1.1 | 56.7 |  |  | 0.4 | 4 | 0.5 | 41.7 | 0.0 | < 10 |  |
| **61** | 1200 | 58 | 1.3 | 50.0 |  |  |  |  |  |  |  |  |  |
| **63** | 1200 | 82 | 1.3 | 48.2 |  |  |  |  |  |  |  |  |  |
| **65** | 1200 | 73 | 1.3 | 49.5 |  |  |  |  |  |  |  |  |  |
| **68** | 1200 |  |  |  |  |  |  |  |  |  |  |  |  |
| **70** | 1200 | 101 | 1.7 | 35.0 |  |  |  |  |  |  |  |  |  |
| **72** | 1200 | 76 | 1.7 | 35.5 |  |  |  |  |  |  |  |  |  |
| **74** | 1200 |  |  |  |  |  |  |  |  |  |  |  |  |
| **76** |  | 58 | 1.5 | 40.5 |  |  | 0.3 | 4 |  |  |  |  |  |
| **77** | 1200 |  |  |  |  |  |  |  |  |  |  |  |  |
| **79** | 1200 | 120 | 1.6 | 36.8 |  |  |  |  |  |  |  |  |  |
| **81** | 1200 | 86 | 1.7 | 35.3 |  |  |  |  |  |  |  |  |  |
| **84** | 1200 | 108 | 2.0 | 29.4 |  |  | 0.2 | 4 |  |  |  |  |  |
| **87** | 1200 |  |  |  |  |  |  |  |  |  |  |  |  |
| **89** | 1200 | 73 | 1.4 | 42.4 |  |  | 0.1 | 4 |  |  |  |  |  |
| **92** | 1200 |  |  |  |  |  |  |  |  |  |  |  |  |
| **94** | 1200 | 69 | 1.8 | 33.4 |  |  |  |  |  |  |  |  |  |
| **96** |  | 72 | 1.5 | 40.9 |  |  | 0.1 | 3 |  |  |  |  |  |
| **97** | 1200 |  |  |  |  |  |  |  |  |  |  |  |  |
| **99** | 1200 |  |  |  |  |  |  |  |  |  |  |  |  |
| **102** | 1200 | 118 | 1.8 | 32.2 |  |  |  |  |  |  |  |  |  |
| **105** | 1200 | 108 | 1.8 | 33.5 |  |  |  |  |  |  |  |  |  |
| **108** | 1200 | 90 | 1.8 | 33.5 |  |  |  |  |  |  |  |  |  |
| **111** | 1200 |  |  |  |  |  | 0.1 | 2 |  |  |  |  |  |

^a^ TP, total serum protein; ^b^ SA, serum albumin; ^c^ hematuria measured by urine dipstic (scale from 0 to 4); ^d^ complement C3 ref. range: 0.9 – 1.8 g/l; ^e^ complement C3d ref. range: < 9 mg/l; ^f^ complement C4 ref. range: 0.1 – 0.4 g/l; ^g^ complement CH50 ref. range: 20 – 50 E/ml; ^h^ sMAC: < 320 ng/ml.

**Additional Table S4. Treatment and response patient *C3GN3***

| **Week** | **Eculizu-**  **mab (mg)** | **BUN (mg/dl)** | **SCr (mg/dl)** | **eGFR (ml/min/**  **1.73m^2^)** | **TP^a^ (g/dl)** | **SA^b^ (g/dl)** | **UPCR (g/g)** | **Hematuria^c^**  **(0-4)** | **C3^d^ (g/l)** | **C3d^e^ (mg/l)** | **C4^f^ (g/l)** | **CH50^g^ (E/ml)** |
| --- | --- | --- | --- | --- | --- | --- | --- | --- | --- | --- | --- | --- |
|  |  |  |  |  |  |  |  |  | 0.46 | 9.1 | 0.08 |  |
| **0** | 900 | 42 | 2.1 | 27.9 |  |  | 0.32 | 3 | 0.47 | 16.3 | 0.12 | 18 |
| **1** | 900 | 46 | 2.0 | 29.5 |  |  | 0.22 | 3 |  |  |  |  |
| **2** | 900 | 60 | 2.5 | 22.9 |  |  | 0.15 | 3 |  |  |  |  |
| **3** | 900 | 48 | 2.1 | 29.0 |  |  | 0.16 | 3 |  |  |  |  |
| **4** | 1200 | 49 | 1.9 | 31.8 |  |  | 0.18 | 4 |  |  |  |  |
| **6** | 1200 | 54 | 1.8 | 33.6 |  |  | 0.16 | 4 |  |  |  |  |
| **8** | 1200 | 56 | 1.9 | 31.6 |  |  |  |  |  |  |  |  |
| **10** | 1200 | 59 | 1.8 | 33.8 |  |  | 0.23 | 4 |  |  |  |  |
| **12** | 1200 | 59 | 2.2 | 27.0 |  |  |  |  |  |  |  |  |
| **14** | 1200 | 55 | 1.9 | 31.6 |  |  | 0.35 | 4 |  |  |  |  |
| **16** | 900 | 52 | 2.0 | 29.5 |  |  |  |  |  |  |  |  |
| **18** | 900 | 57 | 2.0 | 30.1 |  |  | 0.26 | 3 |  |  |  |  |
| **26** | 1200 | 62 | 1.9 |  |  |  |  |  |  |  |  |  |
| **28** | 1200 | 82 | 1.7 | 35.3 |  |  |  |  |  |  |  |  |
| **30** | 1200 | 72 | 2.0 | 28.0 |  |  | 0.11 | 3 |  |  |  |  |
| **32** | 1200 | 50 | 1.8 | 34.1 |  |  |  |  |  |  |  |  |
| **34** | 1200 | 59 | 2.0 |  |  |  | 0.28 | 4 |  |  |  |  |
| **36** | 1200 |  |  |  |  |  |  |  |  |  |  |  |
| **38** | 1200 | 64 | 2.2 | 25.0 |  |  | 0.29 | 4 |  |  |  |  |

^a^ TP, total serum protein; ^b^ SA, serum albumin; ^c^ hematuria measured by urine dipstic (scale from 0 to 4); ^d^ complement C3 ref. range: 0.9 – 1.8 g/l; ^e^ complement C3d ref. range: < 9 mg/l; ^f^ complement C4 ref. range: 0.1 – 0.4 g/l; ^g^ complement CH50 ref. range: 20 – 50 E/ml.

**Additional file Table S5. Treatment and response patient *DDD1***

| **Week** | **Eculizu-**  **mab**  **(mg)** | **BUN (mg/dl)** | **SCr (mg/dl)** | **eGFR (ml/min/**  **1.73m^2^)** | **TP^a^ (g/dl)** | **SA^b^ (g/dl)** | **UPCR (g/g)** | **Hematuria^c^ (0-4)** | **C3^d^ (g/l)** | **C3d^e^ (mg/l)** | **C4^f^ (g/l)** | **CH50^g^ (E/ml)** | **SMAC^h^ (ng/ml)** |
| --- | --- | --- | --- | --- | --- | --- | --- | --- | --- | --- | --- | --- | --- |
| **-34** |  |  |  |  |  |  | 5.7 | 4 | 0,3 | 40,0 |  |  | 3448 |
| **0** | 900 | 59 | 1.5 | 45.7 |  |  |  |  |  |  |  |  |  |
| **1** | 900 | 47 | 1.3 | 55.0 |  |  |  |  |  |  |  |  |  |
| **2** | 900 | 46 | 1.4 | 50.8 |  |  |  |  |  |  |  |  |  |
| **3** | 900 | 52 | 1.3 | 53.0 |  |  | 3.8 | 4* |  |  |  |  |  |
| **5** | 1200 | 66 | 1.3 | 52.1 |  |  |  |  |  |  |  |  |  |
| **7** | 1200 | 60 | 1.2 | 56.0 |  |  | 4.2 | 4* |  |  |  |  |  |
| **9** | 1200 | 53 | 1.3 | 55.5 |  |  |  |  |  |  |  |  |  |
| **11** | 1200 | 54 | 1.4 | 48.7 |  |  |  |  |  |  |  |  |  |
| **15** | 1200 | 57 | 1.4 | 49.9 | 6.1 |  |  |  |  |  |  |  |  |
| **17** | 1200 | 64 | 1.3 | 51.2 |  |  |  |  |  |  |  |  |  |
| **18** |  |  |  |  |  |  |  |  | 0.3 | 40.4 | 0.3 | 21 |  |
| **19** | 1200 | 65 | 1.3 | 54.0 |  |  |  |  |  |  |  |  |  |
| **21** | 1200 | 56 | 1.3 | 51.2 |  |  |  |  |  |  |  |  |  |
| **22** | 1200 | 68 | 1.2 | 56.5 |  |  |  |  |  |  |  |  |  |
| **25** | 1200 | 52 | 1.2 | 60.5 |  |  |  |  |  |  |  |  |  |
| **27** | 1200 | 59 | 1.3 | 55.5 | 6.2 | 3,8 | 2.2 | 1 |  |  |  |  |  |
| **29** | 1200 | 48 | 1.2 | 61.1 |  |  |  |  |  |  |  |  |  |
| **31** | 1200 | 56 | 1.2 | 61.1 |  |  |  |  | 0.2 | 24.9 | 0.2 |  |  |
| **33** | 1200 | 50 | 1.1 | 63.0 |  |  |  |  |  |  |  |  |  |
| **35** | 1200 | 58 | 1.2 | 60.5 | 5.9 |  | 2.5 | 1 |  |  |  |  |  |
| **37** | 1200 | 52 | 1.2 | 56.0 |  |  |  |  |  |  |  |  |  |
| **39** | 1200 | 55 | 1.1 | 64.3 |  |  |  |  |  |  |  |  |  |
| **41** | 1200 | 52 | 1.2 | 61.1 |  |  |  |  |  |  |  |  |  |
| **43** | 1200 | 72 | 1.3 | 51.7 |  |  |  |  |  |  |  |  |  |
| **45** | 1200 | 45 | 1.1 | 61.9 |  |  |  |  |  |  |  |  |  |
| **47** | 1200 | 50 | 1.0 | 68.1 |  |  |  |  |  |  |  |  |  |
| **49** | 1200 | 68 | 1.2 | 60.6 |  |  |  |  |  |  |  |  |  |
| **51** | 1200 | 56 | 1.2 | 58.0 |  |  | 2.7 | 0 |  |  |  |  |  |
| **53** | 1200 | 48 | 1.1 | 64.0 |  |  |  |  |  |  |  |  |  |
| **55** | 1200 | 47 | 1.2 | 58.8 |  |  |  |  |  |  |  |  |  |
| **57** | 1200 | 65 | 1.1 | 61.9 |  |  |  |  |  |  |  |  |  |
| **59** | 1200 | 58 | 1.2 | 57.2 |  |  |  | 0 |  |  |  |  |  |
| **61** | 1200 | 50 | 1.2 | 58.8 |  |  | 2.2 |  |  |  |  |  |  |
| **63** | 1200 | 52 | 1.1 | 65.9 | 5.8 |  |  |  |  |  |  |  |  |
| **65** | 1200 | 55 | 1.1 | 63.8 |  |  |  |  |  |  |  |  |  |
| **67** | 1200 | 68 | 1.0 | 71.2 |  |  |  |  |  |  |  |  |  |
| **69** | 1200 | 54 | 1.2 | 59.4 |  |  |  |  |  |  |  |  |  |
| **71** | 1200 | 68 | 1.4 | 47.5 | 6.4 |  |  |  |  |  |  |  |  |
| **73** | 1200 | 44 | 1.1 | 63.2 |  |  |  |  |  |  |  |  |  |
| **75** | 1200 | 71 | 1.5 | 46.4 |  |  |  |  |  |  |  |  |  |
| **77** | 1200 | 44 | 1.0 | 68.8 |  |  |  |  |  |  |  |  |  |
| **79** | 1200 | 38 | 1.2 | 60.6 |  |  |  |  |  |  |  |  |  |
| **81** | 1200 | 42 | 1.1 | 63.9 |  |  |  |  |  |  |  |  |  |
| **83** | 1200 | 60 | 1.2 | 57.7 |  |  |  |  |  |  |  |  |  |
| **85** | 1200 | 50 | 1.1 | 63.8 |  |  |  |  |  |  |  |  |  |
| **87** | 1200 | 55 | 1.2 | 57.7 |  |  | 3.0 | 0 |  |  |  |  |  |
| **89** | 1200 | 65 | 1.1 | 63.8 |  |  |  |  |  |  |  |  |  |
| **91** | 1200 | 61 | 1.1 | 63.8 |  |  |  |  |  |  |  |  |  |
| **93** | 1200 | 64 | 1.1 | 63.8 |  |  |  |  |  |  |  |  |  |
| **95** | 1200 | 51 | 1.1 | 63.8 |  |  |  |  |  |  |  |  |  |
| **97** | 1200 | 69 | 1.2 | 57.7 |  |  |  |  |  |  |  |  |  |
| **99** | 1200 | 58 | 1.1 | 63.8 | 5.7 | 3.3 | 3.8 | 1 |  |  |  |  |  |

^a^ TP, total serum protein; ^b^ SA, serum albumin; ^c^ hematuria measured by urine dipstic (scale from 0 to 4); ^d^ complement C3 ref. range: 0.9 – 1.8 g/l; ^e^ complement C3d ref. range: < 9 mg/l; ^f^ complement C4 ref. range: 0.1 – 0.4 g/l; ^g^ complement CH50 ref. range: 20 – 50 E/ml; ^h^ sMAC: < 320 ng/ml; * menses.

**Additional file Table S6. Treatment and response patient *C3GN4***

| **Week** | **Eculizu-**  **mab**  **(mg)** | **BUN**  **(mg/dl)** | **SCr**  **(mg/dl)** | **eGFR**  **(ml/min/**  **1.73m^2^)** | **TP^a^**  **(g/dl)** | **SA^b^**  **(g/dl)** | **UPCR**  **(g/g)** | **Hematuria^c^**  **(0-4)** | **C3^d^**  **(g/l)** | **C3d^e^**  **(mg/l)** | **C4^f^**  **(g/l)** | **CH50^g^**  **(E/ml)** |
| --- | --- | --- | --- | --- | --- | --- | --- | --- | --- | --- | --- | --- |
| **0** | 900 | 186 | 3.3 | 26 | 5.2 | 2.9 | 3.4 |  | 0.3 | 35.2 | 0.2 |  |
| **1** | 900 | 108 | 1.8 | 52 | 4.4 | 2.5 | 4.1 |  | 0.3 | 28.3 |  |  |
| **2** | 900 | 134 | 2.0 | 47 | 5.5 | 3.4 | 1.2 |  | 0.3 | 33.1 |  |  |
| **3** | 900 | 133 | 1.9 | 49 | 6.2 | 3.5 | 0.9 |  | 0.2 | 31.9 |  |  |
| **4** | 1200 | 97 | 1.9 | 48 | 6.0 | 3.5 | 1.0 |  | 0.2 | 30.7 |  |  |
| **7** | 1200 | 75 | 1.6 | 58 | 6.2 | 3.5 | 1.1 |  | 0.2 | 22.4 | 0.5 |  |
| **9** | 1200 | 124 | 1.8 | 51 | 6.6 | 3.7 | 0.5 | 4 | 0.2 | 43.6 |  |  |
| **13** | 1200 | 72 | 1.7 | 56 | 6.4 | 3.8 | 1.8 | 4 | 0.2 | 32.0 |  |  |
| **38** |  |  |  |  |  |  |  | 4 | 0.3 | 50.3 | 0.2 | 24.0 |
| **92** | 900 | 186 | 3.0 | 28 |  |  | 4.3 | 4 |  |  |  |  |
| **94** | 900 | 167 | 2.7 | 32 |  |  | 3.8 | 4 |  |  |  |  |
| **95** | 900 | 185 | 2.6 | 34 |  |  |  |  |  |  |  |  |
| **96** | 900 | 158 | 2.7 | 32 |  | 2.5 | 3.6 | 4 |  |  |  |  |
| **97** | 1200 | 168 | 3.0 | 28 |  |  |  |  |  |  |  |  |
| **99** | 1200 | 177 | 3.2 | 26 |  |  | 5.0 | 4 |  |  |  |  |
| **101** | 1200 | 152 | 4.0 | 20 |  | 2.5 | 4.6 | 3 |  |  |  |  |
| **103** | 1200 | 182 | 4.5 | 18 |  |  | 5.7 | 3 |  |  |  |  |

^a^ TP, total serum protein; ^b^ SA, serum albumin; ^c^ hematuria measured by urine dipstic (scale from 0 to 4); ^d^ complement C3 ref. range: 0.9 – 1.8 g/l; ^e^ complement C3d ref. range: < 9 mg/l; ^f^ complement C4 ref. range: 0.1 – 0.4 g/l; ^g^ complement CH50 ref. range: 20 – 50 E/ml.

**Additional file Table S7. Treatment and response patient *DDD2***

| **Week** | **Eculizu-**  **mab**  **(mg)** | **BUN**  **(mg/dl)** | **SCr**  **(mg/dl)** | **eGFR**  **(ml/min/**  **1.73m^2^)** | **TP^a^**  **(g/dl)** | **SA^b^**  **(g/dl)** | **UPCR**  **(g/g)** | **Hematuria^c^**  **(0-4)** | **C3^d^**  **(g/l)** | **C3d^e^**  **(mg/l)** | **C4^f^**  **(g/l)** | **CH50^g^**  **(E/ml)** |
| --- | --- | --- | --- | --- | --- | --- | --- | --- | --- | --- | --- | --- |
| **-4** |  | 142 | 2.1 | 25.8 |  | 3.0 | 5.4 | 4 |  |  |  |  |
| **0** | 900 | 171 | 2.1 | 25.8 |  |  | 5.4 |  | 0.4 | 22.3 | 0.3 | 13 |
| **1** | 900 | 126 | 1.9 | 29.1 |  |  |  |  |  |  |  |  |
| **2** | 900 | 120 | 1.9 | 28.8 |  |  |  |  |  |  |  |  |
| **3** | 900 | 149 | 1.9 | 28.8 |  |  |  |  |  |  |  |  |
| **4** | 1200 | 147 | 2.0 | 26.5 |  | 3.1 | 3.3 | 2 | 0.4 | 30.0 | 0.3 | < 10 |
| **6** | 1200 | 150 | 2.0 | 26.8 |  |  |  |  |  |  |  |  |
| **8** | 1200 | 159 | 1.9 | 28.2 |  | 3.7 | 1.7 | 1 | 0.3 | 47.5 | 0.4 | < 10 |
| **10** | 1200 | 149 | 1.9 | 29.7 |  |  |  |  |  |  |  |  |
| **12** | 1200 | 131 | 1.8 | 30.2 |  |  |  | 2 |  |  |  |  |
| **14** | 1200 | 153 | 2.1 | 25.3 |  |  |  |  |  |  |  |  |
| **16** | 1200 | 147 | 1.9 | 28.1 |  |  |  |  |  |  |  |  |
| **17** |  | 169 | 2.0 | 27.1 |  | 3.4 | 2.2 | 1 | 0.3 | 30.5 | 0.3 | < 10 |
| **18** | 1200 | 159 | 2.0 | 27.6 |  |  |  |  |  |  |  |  |
| **20** | 1200 | 178 | 2.1 | 25.6 |  |  |  |  |  |  |  |  |
| **21** |  | 157 | 1.9 | 28.8 |  | 3.7 | 2.1 | 2 |  |  |  |  |
| **22** | 1200 | 159 | 1.9 | 28.6 |  |  |  |  |  |  |  |  |
| **24** | 1200 | 146 | 2.1 | 26.0 |  |  |  |  |  |  |  |  |
| **26** | 1200 | 134 | 2.2 | 24.9 |  | 3.2 | 5.7 | 3 |  |  |  |  |
| **32** | 1200 | 174 | 2.5 | 21.2 |  |  |  |  | 0.3 | 43.2 | 0.3 | < 10 |
| **34** | 1200 | 188 | 2.7 | 19.4 |  |  |  |  |  |  |  |  |
| **36** | 1200 | 183 | 2.5 | 20.8 |  |  |  |  |  |  |  |  |
| **37** |  | 190 | 2.8 | 18.5 |  | 3.0 | 4.1 | 2 |  |  |  |  |
| **38** | 1200 | 204 | 2.8 | 18.1 |  |  |  |  |  |  |  |  |
| **40** | 1200 | 184 | 3.0 | 17.0 |  |  |  |  |  |  |  |  |
| **42** | 1200 | 185 | 3.2 | 15.5 |  |  |  |  |  |  |  |  |
| **44** | 1200 | 211 | 3.7 | 13.2 |  | 2.7 | 4.2 | 2 | 0.5 |  | 0.4 |  |
| **48** |  |  | start hemo-  dialysis |  |  | 3.1 | 7.1 |  |  |  |  |  |

^a^ TP, total serum protein; ^b^ SA, serum albumin; ^c^ hematuria measured by urine dipstic (scale from 0 to 4); ^d^ complement C3 ref. range: 0.9 – 1.8 g/l; ^e^ complement C3d ref. range: < 9 mg/l; ^f^ complement C4 ref. range: 0.1 – 0.4 g/l; ^g^ complement CH50 ref. range: 20 – 50 E/ml.

**Additional file Table S8. Treatment and response patient *C3GN5***

| **Week** | **Eculizu-**  **mab**  **(mg)** | **BUN**  **(mg/dl)** | **SCr**  **(mg/dl)** | **eGFR (ml/min/**  **1.73m^2^)** | **TP^a^**  **(g/dl)** | **SA^b^**  **(g/dl)** | **UPCR**  **(g/g)** | **Hematuria^c^**  **(0-4)** | **C3^d^**  **(g/l)** | **C3d^e^**  **(mg/l)** | **C4^f^**  **(g/l)** | **CH50^g^**  **(E/ml)** |
| --- | --- | --- | --- | --- | --- | --- | --- | --- | --- | --- | --- | --- |
| **0** | 900 | 24 | 0.8 | 103 | 4.6 | 3 | 5.6 |  | 0.19 | 36.5 | 0.24 |  |
| **1** | 900 | 38 | 0.8 | 109 | 4.7 | 3.2 | 2.2 |  | 0.13 | 32.3 | 0.26 |  |
| **2** | 900 | 34 | 0.7 | 116 | 4.8 | 3.1 | 2.4 |  | 0.13 | 42.7 | 0.22 |  |
| **3** | 900 | 37 | 0.9 | 94 | 5.1 | 3.3 | 1.7 |  | 0.13 | 42.7 | 0.22 |  |
| **4** | 1200 | 35 | 0.8 | 109 | 5.4 | 3.6 | 2.0 |  | 0.13 | 55.3 |  |  |
| **6** | 1200 | 24 | 0.7 | 116 | 6.2 | 4.1 | 3.7 |  | 0.12 | 46.9 |  |  |
| **8** | 1200 | 21 | 0.7 | 131 | 5.7 | 3.5 | 2.8 |  | 0.12 | 61.2 |  |  |
| **9** | 1200 | 31 | 0.6 | 136 | 5.7 | 3.7 | 2.1 |  | 0.13 | 56 |  |  |
| **12** | 1200 | 29 | 0.6 | 133 | 5.7 | 3.7 | 2.1 |  | 0.13 | 70.7 |  |  |
| **14** | 1200 | 21 | 0.6 | 146 | 5.6 | 3.6 | 2.8 |  | 0.14 | 46.1 |  |  |
| **16** | 1200 | 24 | 0.6 | 142 | 5.1 | 3.4 | 3.0 |  | 0.14 | 43.9 |  |  |
| **18** | 1200 | 19 | 0.6 | 145 | 5.4 | 3.6 | 2.8 |  | 0.12 | 28 |  |  |
| **20** | 1200 | 29 | 0.6 | 136 | 5.3 | 3.5 | 3.4 |  |  |  |  |  |
| **22** | 1200 | 19 | 0.6 | 153 | 5.2 | 3.5 | 4.0 | 2 |  | 0.14 |  |  |
| **24** | 1200 | 26 | 0.6 | 153 | 5 | 3.3 | 4.2 |  | 0.16 | 52.3 |  |  |
| **26** | 1200 | 23 | 0.6 | 147 | 5.1 | 3.3 | 4.8 | 2 | 0.2 | 62.1 |  |  |
| **28** | 1200 | 28 | 0.6 | 147 | 5.2 | 3.5 | 3.3 |  |  |  |  |  |
| **32** | 1200 | 21 | 0.5 | 164 | 5.2 | 3.3 | 4.5 |  | 0.18 | 55.8 |  |  |
| **35** | 1200 | 23 | 0.6 | 136 | 5.2 | 3.2 | 2.8 |  | 0.14 | 50 |  |  |
| **37** | 1200 | 25 | 0.6 | 147 | 5.1 | 3.3 | 4.0 |  | 0.18 |  |  |  |
| **40** | 1200 | 23 | 0.6 | 136 | 5.2 | 3.5 | 2.5 |  |  |  |  |  |
| **43** | 1200 | 21 | 0.6 | 134 | 5.2 | 3.5 | 2.5 |  | 0.14 | 70.4 |  |  |
| **47** | 1200 | 20 | 0.6 | 142 | 4.9 |  | 4.3 |  | 0.2 |  |  |  |
| **50** | 1200 | 18 | 0.5 | 160 | 4.8 | 3.2 | 4.4 |  | 0.18 | 54 |  |  |
| **53** | 1200 | 29 | 0.7 | 121 | 4.3 | 2.8 | 3.9 |  | 0.25 | 44.4 |  |  |
| **55** | 1200 | 26 | 0.6 | 142 | 4.5 | 2.9 | 5.5 |  | 0.22 | 44 |  |  |
| **58** | 1200 | 26 | 0.8 | 108 | 4.7 | 2.9 | 4.3 |  | 0.21 | > 75 |  |  |
| **60** | 1200 | 18 | 0.7 | 125 | 4.5 | 2.8 | 3.1 |  | 0.18 | 65.8 |  |  |
| **62** | 1200 | 24 | 0.7 | 129 | 4.8 | 3.2 | 3.0 |  |  |  |  |  |
| **64** | 1200 | 25 | 0.5 | 160 | 5.1 | 3.3 |  |  |  |  |  |  |
| **67** | 1200 | 22 | 0.5 | 164 | 4.4 | 2.5 | 6.3 |  | 0.25 | 75 |  |  |
| **69** | 1200 | 20 | 0.5 | 158 | 4.4 | 2.6 | 9.9 |  | 0.25 |  |  |  |
| **71** | 1200 | 26 | 0.7 | 121 | 4.2 | 2.4 | 5.0 |  | 0.23 | 37.4 |  |  |
| **73** | 1200 | 23 | 0.6 | 137 | 4.1 | 2.6 | 6.9 |  |  |  |  |  |
| **75** | 1200 | 21 | 0.5 | 165 | 4 | 2.5 | 6.4 |  | 0.29 |  |  |  |
| **77** | 1200 | 17 | 0.5 | 165 | 4 | 2.2 | 6.2 |  | 0.22 | 35.2 |  |  |
| **80** | 1200 | 29 | 0.8 | 103 | 3.8 | 2.3 |  |  |  |  |  |  |
| **83** | 1200 | 31 | 0.9 | 94 | 3.7 | 1.9 | 12.3 |  | 0.62 | 33.9 |  |  |
| **85** | 1200 | 42 | 0.8 | 100 | 3.6 | 2.2 | 5.6 |  | 0.58 | 33.3 |  |  |
| **87** | 1200 | 50 | 0.9 | 85 | 3.5 | 1.9 | 6.9 |  | 0.66 | 22 |  |  |
| **89** | 1200 | 34 | 0.9 | 84 | 3.9 | 1.9 | 10.0 |  | 0.66 | 31.2 |  |  |
| **91** | 1200 | 32 | 1.1 | 71 | 3.6 | 1.9 | 11.2 |  |  |  |  |  |
| **92** | 1200 | 29 | 0.7 | 119 | 3.5 | 1.8 | 9.3 |  | 0.35 | 26.8 |  |  |
| **94** | 1200 | 40 | 0.7 | 123 | 3.7 | 2 | 7.7 |  | 0.38 | 41.4 |  |  |
| **95** | 1200 | 36 | 0.8 | 108 | 3.3 | 1.7 | 8.9 |  | 0.42 | 60.2 | 0.28 |  |
| **96** | 1200 | 40 | 0.7 | 110 | 3.9 | 2 | 5.0 |  | 0.41 |  |  |  |
| **97** | 1200 | 33 | 0.7 | 119 | 3.6 | 1.8 | 5.6 |  |  |  |  |  |
| **98** | 1200 | 40 | 0.7 | 113 | 3.5 | 1.9 | 5.6 |  |  |  |  |  |
| **99** | 1200 | 34 | 0.7 | 121 | 3.3 | 1.8 | 5.6 |  | 0.32 | 41.9 | 0.21 |  |
| **101** | 1200 | 42 | 0.8 | 105 | 3.9 | 2 | 6.7 |  | 0.45 | 38.4 |  |  |
| **118** | 1200 | 36 | 1.3 | 59 | 3.2 | 1.6 | 15.8 |  | 0.64 | 26.2 |  |  |
| **120** | 1200 | 39 | 2.0 | 36 | 3.5 | 1.8 | 16.9 | 1 | 0.87 | 22.1 |  |  |

^a^ TP, total serum protein; ^b^ SA, serum albumin; ^c^ hematuria measured by urine dipstic (scale from 0 to 4); ^d^ complement C3 ref. range: 0.9 – 1.8 g/l; ^e^ complement C3d ref. range: < 9 mg/l; ^f^ complement C4 ref. range: 0.1 – 0.4 g/l; ^g^ complement CH50 ref. range: 20 – 50 E/ml.

**References for Supplementary Material**

1. Bresin E, Rurali E, Caprioli J, Sanchez-Corral P, Fremeaux-Bacchi V, Rodriguez de Cordoba S, Pinto S, Goodship TH, Alberti M, Ribes D *et al*: **Combined complement gene mutations in atypical hemolytic uremic syndrome influence clinical phenotype**. *J Am Soc Nephrol* 2013, **24**(3):475-486.

2. Cayci FS, Cakar N, Hancer VS, Uncu N, Acar B, Gur G: **Eculizumab therapy in a child with hemolytic uremic syndrome and CFI mutation**. *Pediatr Nephrol* 2012, **27**(12):2327-2331.

3. Schwarz JM, Cooper DN, Schuelke M, Seelow D: **MutationTaster2: mutation prediction for the deep-sequencing age**. *Nat Methods* 2014, **11**(4):361-362.

4. Adzhubei IA, Schmidt S, Peshkin L, Ramensky VE, Gerasimova A, Bork P, Kondrashov AS, Sunyaev SR: **A method and server for predicting damaging missense mutations**. *Nat Methods* 2010, **7**(4):248-249.

5. Tavtigian SV, Deffenbaugh AM, Yin L, Judkins T, Scholl T, Samollow PB, de Silva D, Zharkikh A, Thomas A: **Comprehensive statistical study of 452 BRCA1 missense substitutions with classification of eight recurrent substitutions as neutral**. *J Med Genet* 2006, **43**(4):295-305.

6. Ferrer-Costa C, Gelpi JL, Zamakola L, Parraga I, de la Cruz X, Orozco M: **PMUT: a web-based tool for the annotation of pathological mutations on proteins**. *Bioinformatics* 2005, **21**(14):3176-3178.

7. Ng PC, Henikoff S: **SIFT: Predicting amino acid changes that affect protein function**. *Nucleic Acids Res* 2003, **31**(13):3812-3814.

8. Skerka C, Jozsi M, Zipfel PF, Dragon-Durey MA, Fremeaux-Bacchi V: **Autoantibodies in haemolytic uraemic syndrome (HUS)**. *Thromb Haemost* 2009, **101**(2):227-232.

9. Jozsi M, Licht C, Strobel S, Zipfel SL, Richter H, Heinen S, Zipfel PF, Skerka C: **Factor H autoantibodies in atypical hemolytic uremic syndrome correlate with CFHR1/CFHR3 deficiency**. *Blood* 2008, **111**(3):1512-1514.

10. Moore I, Strain L, Pappworth I, Kavanagh D, Barlow PN, Herbert AP, Schmidt CQ, Staniforth SJ, Holmes LV, Ward R *et al*: **Association of factor H autoantibodies with deletions of CFHR1, CFHR3, CFHR4, and with mutations in CFH, CFI, CD46, and C3 in patients with atypical hemolytic uremic syndrome**. *Blood* 2010, **115**(2):379-387.

11. Holmes LV, Strain L, Staniforth SJ, Moore I, Marchbank K, Kavanagh D, Goodship JA, Cordell HJ, Goodship TH: **Determining the population frequency of the CFHR3/CFHR1 deletion at 1q32**. *PLoS One* 2013, **8**(4):e60352.

12. Bomback AS, Smith RJ, Barile GR, Zhang Y, Heher EC, Herlitz L, Stokes MB, Markowitz GS, D'Agati VD, Canetta PA *et al*: **Eculizumab for dense deposit disease and C3 glomerulonephritis**. *Clin J Am Soc Nephrol* 2012, **7**(5):748-756.

13. Le Quintrec M, Lionet A, Kandel C, Bourdon F, Gnemmi V, Colombat M, Goujon JM, Fremeaux-Bacchi V, Fakhouri F: **Eculizumab for treatment of rapidly progressive C3 glomerulopathy**. *Am J Kidney Dis* 2015, **65**(3):484-489.

14. Daina E, Noris M, Remuzzi G: **Eculizumab in a patient with dense-deposit disease**. *N Engl J Med* 2012, **366**(12):1161-1163.

15. Vivarelli M, Pasini A, Emma F: **Eculizumab for the treatment of dense-deposit disease**. *N Engl J Med* 2012, **366**(12):1163-1165.

16. McCaughan JA, O'Rourke DM, Courtney AE: **Recurrent dense deposit disease after renal transplantation: an emerging role for complementary therapies**. *Am J Transplant* 2012, **12**(4):1046-1051.

17. Gurkan S, Fyfe B, Weiss L, Xiao X, Zhang Y, Smith RJ: **Eculizumab and recurrent C3 glomerulonephritis**. *Pediatr Nephrol* 2013, **28**(10):1975-1981.

18. Kerns E, Rozansky D, Troxell ML: **Evolution of immunoglobulin deposition in C3-dominant membranoproliferative glomerulopathy**. *Pediatr Nephrol* 2013, **28**(11):2227-2231.

19. Rousset-Rouviere C, Cailliez M, Garaix F, Bruno D, Laurent D, Tsimaratos M: **Rituximab fails where eculizumab restores renal function in C3nef-related DDD**. *Pediatr Nephrol* 2014, **29**(6):1107-1111.

20. Ozkaya O, Nalcacioglu H, Tekcan D, Genc G, Meydan BC, Ozdemir BH, Baysal MK, Keceligil HT: **Eculizumab therapy in a patient with dense-deposit disease associated with partial lipodystropy**. *Pediatr Nephrol* 2014, **29**(7):1283-1287.

21. Berthe-Aucejo A, Sacquepee M, Fila M, Peuchmaur M, Perrier-Cornet E, Fremeaux-Bacchi V, Deschenes G: **Blockade of alternative complement pathway in dense deposit disease**. *Case Rep Nephrol* 2014, **2014**:201568.

22. Sanchez-Moreno A, De la Cerda F, Cabrera R, Fijo J, Lopez-Trascasa M, Bedoya R, Rodriguez de Cordoba S, Ybot-Gonzalez P: **Eculizumab in dense-deposit disease after renal transplantation**. *Pediatr Nephrol* 2014, **29**(10):2055-2059.

23. Tran CL, Sethi S, Murray D, Cramer CH, Sas DJ, Willrich M, Smith RJ, Fervenza FC: **Discontinuation of dialysis with eculizumab therapy in a pediatric patient with dense deposit disease**. *Pediatr Nephrol* 2016, **31**(4):683-687.

24. Oosterveld MJ, Garrelfs MR, Hoppe B, Florquin S, Roelofs JJ, van den Heuvel LP, Amann K, Davin JC, Bouts AH, Schriemer PJ *et al*: **Eculizumab in Pediatric Dense Deposit Disease**. *Clin J Am Soc Nephrol* 2015, **10**(10):1773-1782.

25. Lebreton C, Bacchetta J, Dijoud F, Bessenay L, Fremeaux-Bacchi V, Sellier-Leclerc AL: **C3 glomerulopathy and eculizumab: a report on four paediatric cases**. *Pediatr Nephrol* 2017.

26. Payette A, Patey N, Dragon-Durey MA, Fremeaux-Bacchi V, Le Deist F, Lapeyraque AL: **A case of C3 glomerulonephritis successfully treated with eculizumab**. *Pediatr Nephrol* 2015, **30**(6):1033-1037.

27. Inman M, Prater G, Fatima H, Wallace E: **Eculizumab-induced reversal of dialysis-dependent kidney failure from C3 glomerulonephritis**. *Clin Kidney J* 2015, **8**(4):445-448.

28. Besbas N, Gulhan B, Gucer S, Korkmaz E, Ozaltin F: **A novel CFHR5 mutation associated with C3 glomerulonephritis in a Turkish girl**. *J Nephrol* 2014, **27**(4):457-460.
